# Do High Volume anti Dementia drug prescribing doctors bill Medicare for Care Plans?

**DOI:** 10.1093/geroni/igaf122.3917

**Published:** 2025-12-31

**Authors:** Michael Splaine, Kate Gordon

**Affiliations:** Splaine Consulting, Columbia, Maryland, United States; Splaine Consulting, Columbia, Maryland, United States

## Abstract

Medical providers can bill the Centers for Medicare and Medicaid Services (CMS) for dementia care plans under code 99483. Dementia care plans provide written information to help provide education and support to the patient and their care provider. The purpose of this data analysis was to determine the relationship between high-volume dementia medication prescribers to providers of dementia care plans. Identifying the relationship between these two factors may help shine a light on a way to improve care for individuals with dementia. The billing codes analyzed were for dementia care plans by high-volume dementia prescription providers across five U.S. markets states from the 2021 Centers for Medicare and Medicaid Services (CMS) database. The main finding across all five was that there was a significantly low use (5% or less) of the dementia care plan billing code amongst the high-volume dementia medication prescribers.

